# Longitudinal associations between white matter maturation and cognitive development across early childhood

**DOI:** 10.1002/hbm.24690

**Published:** 2019-06-12

**Authors:** Xiongtao Dai, Pantelis Hadjipantelis, Jane‐Ling Wang, Sean C. L. Deoni, Hans‐Georg Müller

**Affiliations:** ^1^ Department of Statistics Iowa State University Ames Iowa; ^2^ Department of Statistics University of California Davis Davis California; ^3^ Advanced Baby Imaging Lab Brown University School of Engineering Providence Rhode Island; ^4^ Children's Hospital Imaging of Learning & Development Lab, Department of Radiology University of Colorado School of Medicine, Anschutz Medical Campus Aurora Colorado

**Keywords:** brain MRI, cognitive development, concurrent regression modeling, infant brain development, myelination

## Abstract

From birth to 5 years of age, brain structure matures and evolves alongside emerging cognitive and behavioral abilities. In relating concurrent cognitive functioning and measures of brain structure, a major challenge that has impeded prior investigation of their time‐dynamic relationships is the sparse and irregular nature of most longitudinal neuroimaging data. We demonstrate how this problem can be addressed by applying functional concurrent regression models (FCRMs) to longitudinal cognitive and neuroimaging data. The application of FCRM in neuroimaging is illustrated with longitudinal neuroimaging and cognitive data acquired from a large cohort (*n* = 210) of healthy children, 2–48 months of age. Quantifying white matter myelination by using myelin water fraction (MWF) as imaging metric derived from MRI scans, application of this methodology reveals an early period (200–500 days) during which whole brain and regional white matter structure, as quantified by MWF, is positively associated with cognitive ability, while we found no such association for whole brain white matter volume. Adjusting for baseline covariates including socioeconomic status as measured by maternal education (SES‐ME), infant feeding practice, gender, and birth weight further reveals an increasing association between SES‐ME and cognitive development with child age. These results shed new light on the emerging patterns of brain and cognitive development, indicating that FCRM provides a useful tool for investigating these evolving relationships.

## 1 INTRODUCTION

The first 1,000 days of life, spanning conception to a child's second birthday, represent an important period during which the foundations for the lifelong development of brain and cognition are established (Pujol et al., 2006; Räikkönen, Pesonen, Roseboom, & Eriksson, 2012). Across this age range, the brain's structural and functional growth are at their most rapid (Dubois et al., 2008, 2014; Johnson, 2001; Lenroot & Giedd, 2006). Advancements in magnetic resonance imaging (MRI) techniques have allowed brain structure and function to be mapped and characterized across early childhood (Fair et al., 2007; Gilmore et al., 2012; Knickmeyer et al., 2008).

Previous analyses have revealed early and persistent cross‐sectional and longitudinal differences in brain structure, volume, and growth associated with cognitive ability (Deoni et al., 2016; O'Muircheartaigh et al., 2014), infant feeding and nutrition (i.e., breast milk vs. formula vs. mixed feeding) (Deoni et al., 2013), specific genotypes (Dean et al., 2014; Knickmeyer et al., 2014), and measures of child and family socioeconomic status (SES; Noble et al., 2015). Especially the influence of early nutrition and feeding choice has received significant attention (Anderson, Johnstone, & Remley, 1999; Girard, Doyle, & Tremblay, 2017; Horwood & Fergusson, 1998; Huang, Peters, Vaughn, & Witko, 2014; Kramer et al., 2008; Lucas, Morley, Cole, Lister, & Leeson‐Payne, 1992; Mortensen, Michaelsen, & Sanders, 2002), and previous cross‐sectional neuroimaging studies have revealed differences in brain structure in infancy, childhood, and adolescence in breast versus formula‐fed children, including differences in regional and total brain white and gray matter volume (Deoni, Dean, et al., 2013; Isaacs et al., 2010; Kafouri et al., 2013; Luby, Belden, Whalen, Harms, & Barch, 2016; Ou et al., 2014).

There is, however, less known about the time‐evolution of the relationship between brain structure and cognitive development, which was the primary motivation for our study. The nature of the co‐development of these two longitudinal processes has been of recent interest (Girault et al., 2019; Jolles et al., 2016). Previous modeling of the relationship between white matter and cognitive development is sparse and has been cross‐sectional (Nagy, Westerberg, & Klingberg, 2004), considered a few age bins (Dubois et al., 2008; Jolles et al., 2016), or used parametric random effects models to provide longitudinal summaries for trajectory clustering (Deoni et al., 2016). As these studies used coarse‐grained methods to deal with the longitudinal aspects, they did not go much beyond establishing a general positive relationship between cognitive and structural brain development.

In the present study, our focus was on white matter maturation as quantified by myelin water fraction (MWF), and we also considered whole brain white matter volume. Both metrics were extracted from longitudinal MRI measurements (Deoni et al., 2011). While MWF has previously been shown to correlate with cognitive and behavioral development (Deoni, Dean III, Joelson, O'Regan, & Schneider, 2018; Fields, 2008; Fields, 2010; Nagy et al., 2004; Zatorre, Fields, & Johansen‐Berg, 2012), the nature of this relationship is still largely unknown, which motivated our study. Specifically, we analyzed data from a longitudinal study of 210 children between 65 and 1,481 postnatal days to elucidate the time‐evolving relationships between language, motor, and general cognitive functioning, derived from the Mullen Scales of Early Learning (MSEL, Mullen, 1995), and concurrent measures of white matter MWF, obtained using a multicomponent relaxometry approach (Deoni et al., 2016; MacKay et al., 1994). The MSEL is a standardized and population‐normed assessment tool for measuring emerging thinking skills, language, and motor development in children from birth to 68 months of age.

Myelination is a fundamental process of early development. Beginning in the cerebellum and brainstem, the elaboration of the myelin sheath around neuronal axons follows a characteristic posterior‐to‐anterior, deep‐to‐superficial arc (Barkovich, Kjos, Jackson, & Norman, 1988; Paus et al., 2001; Yakovlev & Lecours, 1967). This pattern of development spatially and temporally mirrors maturing cognitive abilities (Nagy et al., 2004). That is, increases in language ability are associated with the maturity of language networks and brain regions supporting this skill, due to the tight coupling between myelination and neural activities (Fields, 2005; Fields & Stevens‐Graham, 2002).

A second major goal of our study was to demonstrate how one can address the problem of sparsely and irregularly observed longitudinal measurements that is prevalent in many brain developmental studies. The longitudinal modeling approach we proposed addresses the complexity of sparsely observed longitudinal data as present in our cohort. Here applied to white matter structural development, the proposed models can also be widely utilized to study other developmental processes, such as longitudinal cortical maturation or morphometry through different imaging measurements.

Previously, parametric models for longitudinal data have typically been used to investigate differences in the shape and pattern of early brain developmental trajectories (Remer et al., 2017). Depending on the imaging measure and the investigated age range, both linear and nonlinear parametric models coupled with mixed effects have been used. This ranges from fitting linear, quadratic, and cubic trajectories (for cortical thickness and surface area, depending on region; Shaw et al., 2008), to modeling curvilinear associations such as logarithmic (total and regional brain volumes), exponential (quantitative relaxation times and diffusion imaging metrics including fractional anisotropy, qT_1_, qT_2_, FA, and the axial and radial diffusivities, AD and RD; Hasan, 2013; Lebel & Beaulieu, 2011), inverted U‐pattern (Arshad, Stanley, & Raz, 2016), and sigmoidal relationships (white matter myelination, Dean, Jerskey, et al., 2014; Croteau‐Chonka et al., 2016).

While parametric models provide an important basis for longitudinal modeling and the fitted model parameters as metrics can exhibit child group differences or correlate with demographic or cognitive variables of interest, they suffer from a lack of flexibility and require substantial prior knowledge in order to avoid biases. These models may not transfer across brain regions or pediatric populations and are often difficult to validate. In contrast, nonparametric modeling techniques from functional data analysis (Chen, Zhang, Petersen, & Müller, 2017; Müller, 2008; Ramsay & Silverman, 2005; Wang, Chiou, & Müller, 2016) can be advantageous since they make no a priori assumption regarding the shape or structure of data, but instead learn adequate flexible shapes from the data. Additionally, the reconstruction of continuous trajectories using parametric models is complicated by the sparse, unbalanced, and noisy nature of typical longitudinal MRI and neurocognitive data, which is often due to logistical challenges encountered in the majority of developmental imaging studies.

Prior parametric analyses typically involved examining sequential cross‐sectional relationships; group‐wise comparisons of structural trajectories in children stratified by cognitive ability; or associations between changing cognitive score and changing brain structure across variable age windows. While informative, these methodologies provide an incomplete and often fragmented view of the evolving relationships between brain structure and cognition. One goal of our study was to demonstrate that these limitations can be addressed by employing a functional data analysis methodology to construct FCRMs (Şentürk & Müller, 2010), where measures of cognitive functioning are directly related to concurrent measures of brain structure. FCRM is able to handle sparse and irregular longitudinal observations, which is realistic and common in longitudinal neuroimaging studies. Moreover, FCRM is highly flexible, making it the ideal tool for discovering the shape of the underlying developmental trajectories when there is insufficient prior knowledge to adopt parametric models.

Imaging methods, including quantitative T_1_ and T_2_ relaxometry, diffusion (tensor and higher order models), magnetization transfer, and susceptibility‐weighted imaging each inform on complementary aspects of white matter microstructure and myelin content (Alexander et al., 2011). Multicomponent relaxometry (MCR) is a method that decomposes the measured tissue MRI signal into contributions from distinct microanatomical water pools based on their relaxation properties (MacKay et al., 2006). MCR consistently reveals the presence of at least two water pools within brain tissue. Human disease and histological (Laule et al., 2006) studies have ascribed the two pools to restricted myelin water trapped between the lipid bilayers of the myelin sheath, and the less restricted intracellular and extracellular water (MacKay et al., 1994). MWF, defined as the fractional ratio of these two pools, provides a validated and noninvasive assessment of myelin density that well correlates with histological assessments (Kolind et al., 2012; Laule et al., 2006; Wood et al., 2016). However, few previous studies have applied MWF imaging to study pediatric populations, or more broadly, neurodevelopment. This reflects both the relatively recent development of whole‐brain MWF imaging methods (Deoni, Rutt, Arun, Pierpaoli, & Jones, 2008) and the difficulty in imaging pediatric populations.

To obtain whole‐brain MWF measures in a time‐efficient protocol, we use the multicomponent DESPOT (mcDESPOT, Deoni et al., 2008; Deoni, Dean, O'Muircheartaigh, Dirks, & Jerskey, 2012) method. We have previously used mcDESPOT MCR to characterize the spatiotemporal pattern of human brain myelination as reflected by MWF (Deoni et al., 2012; Deoni et al., 2016), and to explore the cross‐sectional relationships between MWF and cognitive development (Deoni et al., 2016; O'Muircheartaigh et al., 2014). In addition to pediatric applications, mcDESPOT has also been used to investigate myelin change in known white matter demyelinating and dysmyelinating disorders, for example, multiple sclerosis (Kitzler et al., 2012; Kolind et al., 2012; Kolind et al., 2013), Alzheimer's disease (Dean et al., 2017), and dementia (Bouhrara et al., 2018).

In this work, we sought to identify critical growth periods when white matter myelination as measured by MWF has significant associations with cognitive function. FCRM is employed on longitudinal MSEL and white matter MWF data acquired from a large cohort (*n* = 210) of healthy and typically developing children spanning 2 months to 4 years of age, adjusting for baseline covariates including SES as measured by maternal education (SES‐ME), infant feeding choice (breastmilk, formula, or mixed feeding), child gender, and birth weight. Our results reveal that white matter MWF is positively associated with cognitive ability in an early developmental period (200–500 days) and that breast feeding, female child, higher SES‐ME, as well as increased birth weight, are associated with better cognitive abilities, where the association of SES‐ME increases with child age. Additional analysis suggests that MWF in different brain regions has differing strength of association with cognitive abilities, which also varies temporally over developmental periods, thus providing for the first time an assessment of the spatiotemporal relations between brain structural and cognitive development.

## 2 METHODS

### 2.1 Population demographics

**Figure 1:**
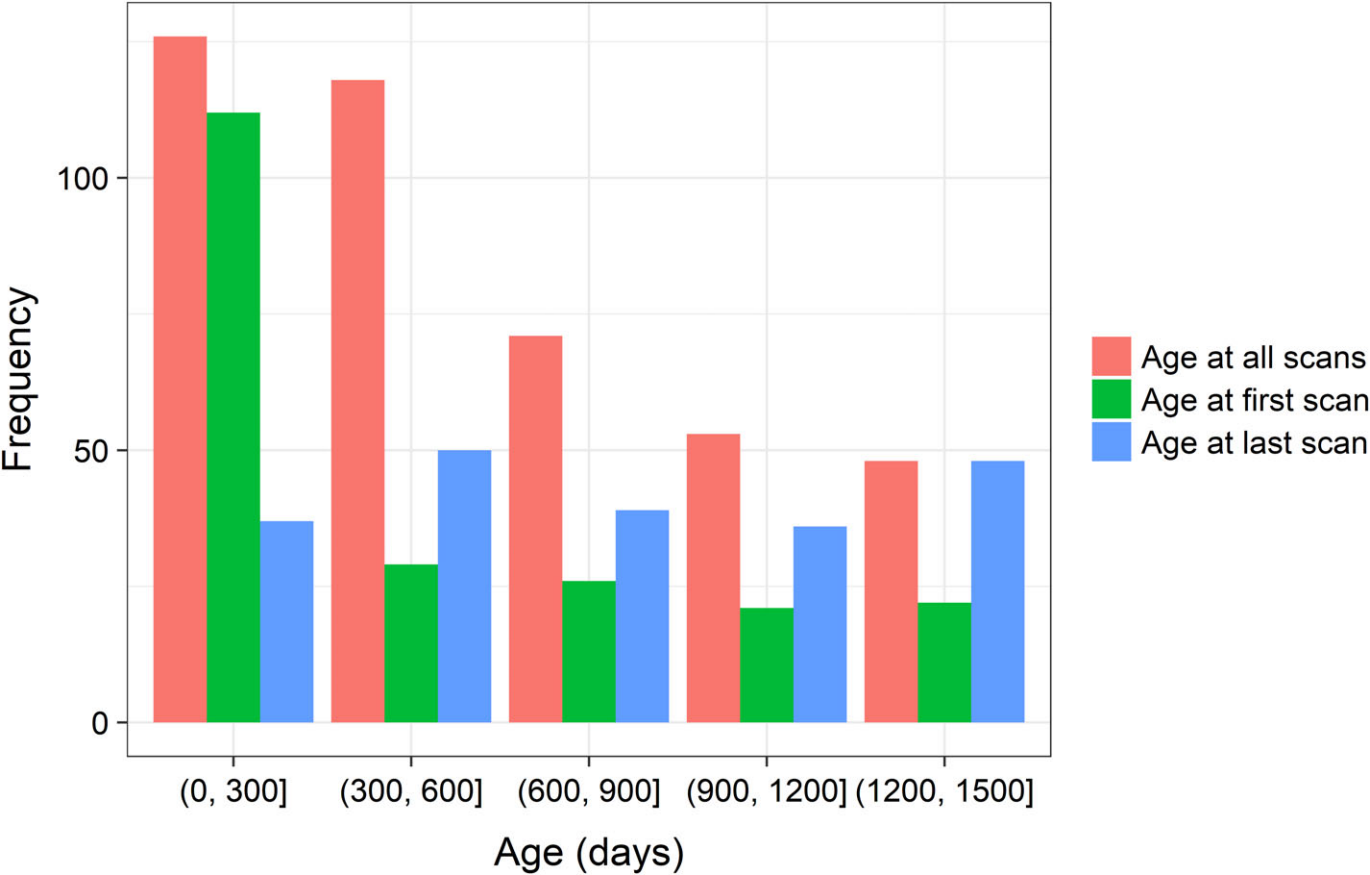
Distributions for age‐at‐all‐scans combined (red), age at first scan (green), and age‐at‐last‐scan (blue) [Color figure can be viewed at http://wileyonlinelibrary.com]

**Figure 2:**
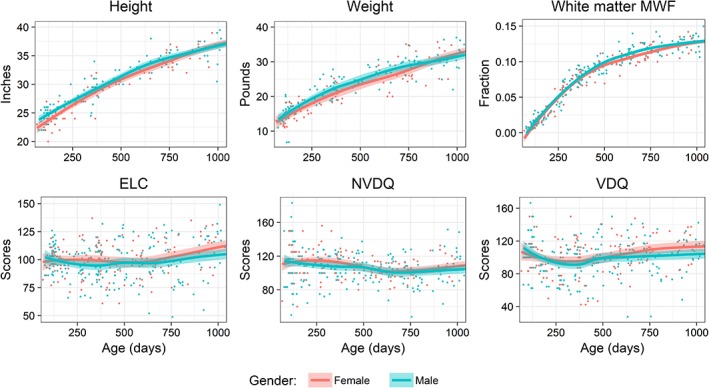
Longitudinal growth measurements (height, weight, whole brain white matter MWF) and cognitive scores (ELC, NVDQ, and VDQ) made at scans. The bold curves are the estimated mean curves over time by local quadratic smoothing, and the shaded bands are 95% confidence intervals (very narrow for white matter MWF) [Color figure can be viewed at http://wileyonlinelibrary.com]

Four hundred and sixteen longitudinal data points from a total of 210 children (120 male) recruited as part of a large study of neurotypical development (the BAMBAM study) were included in this analysis. The age‐range of acquired data spans 65–1,481 postnatal days, corrected for a 40‐week gestation duration. Among all children, 93 were scanned once; 60 were scanned two times; 30 were scanned three times; 23 were scanned four times; 3 were scanned five times; and 1 was scanned six times. In general, children under 2 years of age were scheduled to have follow‐up visits (including MRI and psychometric assessments) every 6 months; and children over 2 years were followed annually. The distributions of age‐at‐all‐scans (pooling all scans for all children), age‐at‐first‐scan, and age‐at‐last‐scan are reported in Figure 1, which shows most of the scans were made before 900 days of age, with more scans available within the younger age range. Most of the children had their first scans around 180 days of age, though some had their first scans after 1,000 days (and thus providing limited information for our analysis); most scans were made around 250 days of age. A summary of the typically developing sample is provided in Table 1, and a display of longitudinal growth and cognitive measurements by gender is shown in Figure 2.

**Table 1:** Study population demographics for the whole sample

Gender	
Male (*n*)	120
Female (*n*)	90
Racial background	
Caucasian (*n*)	114
African American (*n*)	26
Asian (*n*)	6
Hispanic (*n*)	22
Mixed race (*n*)	42
Parent marital status	
Married/living together (*n*)	162
Divorced/single (*n*)	48
Number of times scanned	1×:2×:3×:4×+ 93:60:30:27
Age‐at‐all‐scans	584.1 ± 402.0
Age‐at‐first‐scan	473.6 ± 423.9
Age‐at‐last‐scan	753.2 ± 431.2
Number of children in family (*n*)	2.1 ± 1.2
Gestation (weeks)	39.5 ± 1.3
Birth weight (g)	3,350 ± 487
Birth length (cm)	51 ± 3.06
Maternal education	5.74 ± 1.14
Paternal education	5.70 ± 1.08
ELC	101.0 ± 16.7
NVDQ	108.3 ± 17.9
VDQ	101.5 ± 22.0

Children for this study came from a larger longitudinal study of normal brain development (Deoni et al., 2012) and were recruited from Providence, Rhode Island, and the surrounding areas. To date, approximately 470 children have been recruited between the ages of 1 month and 5 years of age with study visits performed at 6‐ or 12‐month increments. Children with known risk factors for abnormal brain or cognitive development were excluded. These factors included in utero exposure to alcohol, cigarette smoke, or other illicit substances; premature birth before 37 weeks' gestation, neurological trauma, or family history of major psychiatric or learning disorder, including maternal depression requiring medication. Specific inclusion criteria included: (a) healthy singleton birth between 37 and 42 weeks' gestation; (b) uncomplicated pregnancy and delivery; (c) APGAR scores >8; (d) no reported abnormalities on fetal ultrasound; (e) no reported neurological history in the child; (f) No reported psychiatric or learning disability history in the child or first degree relatives. Inclusion criteria were confirmed during phone interview prior to enrollment. Infant, parent, and sibling history questionnaires were used to verify inclusion criteria as well as gather additional information regarding each child's neurological and psychiatric history; maternal and paternal education levels; maternal prenatal and postnatal health, substance use, and breastfeeding practices; gestation duration; and birth weight. Maternal SES was determined using the Hollingshead 4‐Factor Index (HI; Hollingshead, 1975). Specifically, we used the 7‐point educational index to reflect overall socioeconomic status, where 1 corresponds to less than a 7th grade education; 2 to junior high school; 3 to partial high school; 4 to high school graduate; 5 to at least 1 year of college or university; 6 to college or university graduate; and 7 to a professional or graduate degree. Our analysis included only data records that have a complete set of considered variables.

Written informed consent was obtained from each child's parents or legal guardian and the study was performed with approval from the Brown University Internal Review Board.

### 2.2 MRI methods

Children under 4 years of age were, in general, scanned during natural, nonsedated sleep; while children over this age who could remain still were scanned while watching a favorite movie. All imaging was performed on a 3‐Tesla Siemens Tim Trio scanner equipped with a 12‐channel head RF array. To minimize intra‐scan motion, children were swaddled with an infant or pediatric MedVac vacuum immobilization bag (CFI Medical Solutions, Fenton, MI) and foam cushions were placed around their head. Scanner noise was reduced by limiting the peak gradient amplitudes and slew‐rates to 25 mT/m/s. A noise‐insulating insert (Quiet Barrier HD Composite, UltraBarrier, San Leandro, CA) was also fitted to the inside of the scanner bore. MiniMuff pediatric ear covers and electrodynamic headphones (MR Confon, Germany) were used for all children. A pediatric pulse‐oximetry system and infrared camera were used to continuously monitor the infants and children during scanning (Dean, Jerskey, et al., 2014).

#### 2.2.1 mcDESPOT imaging

Age‐specific and acoustically muffled imaging protocols (Deoni et al., 2012) were used to acquire quantitative qT_1_, qT_2_ and MWF data in each subject using the mcDESPOT method (Deoni et al., 2008). Each mcDESPOT protocol consisted of 8 T_1_‐weighted spoiled gradient echo images (SPGR or spoiled FLASH) and 16 balanced T_1_/T_2_‐weighted steady‐state free precession (bSSFP or TrueFISP) images acquired across multiple flip angles (Deoni, Matthews, & Kolind, 2013). Two inversion‐prepared (IR) SPGR images were additionally acquired for correction of radiofrequency (B_1_) inhomogeneities and bSSFP images were acquired with two‐phase cycling patterns (180° and 0°) for correction of main magnetic field (B_0_) inhomogeneities (Deoni, 2011). Total imaging times ranged from 15 minutes for the youngest infants to 24 minutes for older children. Imaging protocols are provided in Table 2. In all cases, the spatial resolution was held constant, with the field of view and imaging matrix increased to accommodate changing child head size.

**Table 2:** Age‐optimized mcDESPOT protocols

Age group (months)	3–9	9–16	16–28	28–48	48+
Field of view (cm^3^)	14 × 14 × 13	17 × 17 × 14.4	18 × 18 × 15	20 × 20 × 15	20 × 20 × 16.5
Acquisition matrix	80 × 80 × 72	96 × 96 × 80	100 × 100 × 88	112 × 112 × 88	112 × 112 × 96
SPGR TE/TR (ms)	5.8/12	5.9/12	5.4/12	5.2/11	4.8/10
SPGR Flip angles (degrees)	2, 3, 4, 5, 7, 9, 11, 14	2, 3, 4, 5, 7, 9, 11, 14	2, 3, 4, 5, 7, 9, 11, 14	2, 3, 4, 5, 7, 9, 12, 16	3, 4, 5, 6, 7, 9, 13, 18
IR‐SPGR inversion times (ms)	600, 950	600, 900	500, 850	500, 800	450, 750
bSSFP TE/TR (ms)	5/10	5.1/10.2	5/10	4.4/8.8	5/10
bSSFP Flip angles (degrees)	9, 14, 20, 27, 34, 41, 56, 70	9, 14, 20, 27, 34, 41, 56, 70	9, 14, 20, 27, 34, 41, 56, 70	9, 14, 20, 27, 34, 41, 56, 70	9, 14, 20, 27, 34, 41, 56, 70

Following acquisition, data were visually assessed for motion artifacts (including blurring, ghosting, etc.) by the same research team member (SCLD) and standard mcDESPOT processing was performed (Deoni et al., 2012). Approximately 5% of all data (22 scans) acquired had significant motion‐related artifacts and was deemed unusable. In addition to visible artifacts, an automated approach was used that flagged participants that displayed more than 2 mm of mean motion in the center of gravity between each SPGR and bSSFP image. No additional data were discarded on the basis of this automated metric. Standard mcDESPOT processing included linear co‐registration of the individual's SPGR, IR‐SPGR, and bSSFP images to account for potential intra‐scan head movement (Jenkinson, Bannister, Brady, & Smith, 2002), nonparenchymal voxel removal (Smith, 2002), and correction of flip angle (B1) errors and off‐resonance (B0) inhomogeneities using DESPOT1‐HIFI and DESPOT2‐FM (Deoni, 2011). From these preprocessed data, voxel‐wise MWF estimates were calculated using a three‐pool model, representing the myelin‐associated water, intra/extracellular water, and a nonexchanging free water pool corresponding to cerebral spinal fluid. This model expands on the two‐pool model (Deoni et al., 2008) and incorporates additional nonexchanging components into the matrices of steady‐state SPGR and bSSFP magnetizations, relative water pool volume fraction, relaxation, off‐resonance, exchange rate, and excitation flip angle; for details, see Deoni, Matthews, and Kolind (2013). A stochastic region contraction fitting approach was used to fit the model to the SPGR and bSSFP data. These quantitative images (“maps”) were then nonlinearly aligned to a custom common analysis space in approximate MNI space using a previously described multistep approach (Deoni et al., 2012) that first aligns the subject's high flip angle T_1_ weighted SPGR image to an age‐specific template and then applies the calculated transformation matrix to the quantitative maps. All image registration/normalization was performed using the ANTS tools (Avants et al., 2011). We have previously shown that this approach provides robust registration across the investigated age range (2–68 months) without altering the quantitative values (Dean III et al., 2014).

### 2.3 Neuropsychological assessments

For all children, cognitive functioning was assessed using the Mullen Scales of Early Learning, MSEL, an assessment enjoying high test–retest reliability (Mullen, 1995). The battery consists of 144 items that are equally distributed across five main sub‐tests: Expressive and receptive language, visual reception, and fine and gross motor function. Normalized composite scores, including the early learning composite (ELC), and verbal and nonverbal development quotients (VDQ and NVDQ, respectively) reflect overall cognitive, language, and motor functioning, respectively. Each of these normalized measures has a mean of 100 and standard deviation (*SD*) of 15. VDQ covers test items of expressive and receptive language; NVDQ comprises test items measuring visual reception, fine motor, and gross motor function.

All cognitive assessments were performed within 7 days of successful MRI by one of three qualified raters trained and supervised by the same licensed clinical neuropsychologist. Assessments were performed using the same standardized stimuli in a consistent testing environment.

### 2.4 Functional concurrent regression models

We examined the time‐dynamic concurrent association between cognitive composite scores (ELC, VDQ, and NVDQ) and white matter maturation (measured by MWF) at different ages by means of FCRMs, which are also referred to as functional varying coefficient models (Şentürk & Müller, 2010). As the measurements available for each child were extremely sparse and irregular over the age range, time‐dynamic regression modeling is a challenging task. Owing to the irregularity of the data in time, with generally few data points acquired at any one age or time‐point, modeling cannot be approached simply by fitting models cross‐sectionally at each time‐point. Further, estimating the time‐dynamic models using a sliding window approach, or by grouping, the longitudinal data in large age windows may disregard important developmental differences occurring within each window, and as a result, may incur additional biases if appropriate adjustments are not made. This may be particularly true for early brain development and myelination as measured by MWF, which occurs rapidly over the first 2–4 years (Deoni et al., 2012; Dean et al., 2014; Yakovlev & Lecours, 1967).

As an alternative approach, FCRMs are well‐established in the statistical literature and have been extended to the case of sparse and irregular observations (Şentürk & Müller, 2010), and subsequently extended further (Şentürk & Nguyen, 2011) to incorporate multiple longitudinal and baseline covariates. We assume that the longitudinal covariates and the response have underlying smooth trajectories over time *t* ∈ *I*, where *I* is the time interval of interest. For each child, we observed the longitudinal covariates and the response in his/her sporadic visits. We denote the response by *Y*(*t*), the *k*th longitudinal covariate for *k* = 1, … , *p* by *X*_*k*_(*t*), furthermore the *l*th baseline covariate by *Z*_*l*_ for *l* = 1, … , *q*, and let **W**(*t*) = (X_1_(*t*),  … , *X*_*p*_(*t*), *Z*_1_,  … , *Z*_*q*_)′ be a column vector containing all covariates. Following Şentürk and Nguyen (2011), the FCRM is given by

(1) $$E\left(Y\left(t\right)|W\left(t\right)\right)=\alpha \left(t\right)+\beta {\left(t\right)}^{\prime }W\left(t\right),$$

which we expand to

(2) $$E\left(Y\left(t\right)|W\left(t\right)\right)=\alpha \left(t\right)+\sum_{k=1}^{p}\beta_{k}\left(t\right)X_{k}\left(t\right)+\sum_{l=1}^{q}\beta_{p+l}\left(t\right)Z_{l},$$

where *α*(*t*) is the intercept, and **β**(t) = (*β*_1_(*t*),  … , *β*_*p* + *q*_(*t*))′ is the vector of regression coefficients, for which the first *p* entries correspond to longitudinal covariates (X_1_(*t*),  … , *X*_*p*_(*t*)) and the last *q* entries to baseline covariates (*Z*_1_,  … , *Z*_*q*_). The response *Y*(*t*) depends linearly on the longitudinal and baseline covariates **W**(*t*) at age *t*, where the effects are reflected in the regression coefficients **β**(t). The coefficients, therefore, characterize the time‐dynamics of the association between the response and the covariates.

The FCRM allows for arbitrary smooth changes in **β**(t) as age varies and, therefore, is considerably more flexible than any linear or other random effects model (e.g., Gautam et al., 2014). For a fixed age *t*_0_, **β**(t_0_) can be interpreted in the same way as the regression parameters in a linear regression model, relating the effects of *X*_k_(*t*) and *Z*_v_ to the response *Y*(*t*). We emphasize here that for most times *t*, none of these covariates or responses are actually observed, which motivates the application of functional data analytic methods whereby one gains strength by borrowing information across the sample.

The estimation procedures are carried out by kernel smoothing. Further details about the estimation method are presented in the Appendix. We note that the intercept and the slop functions can be consistently estimated by borrowing information across subjects, even if only sparse measurements on each subject are available. The software implementation of our procedure (FCReg) is included in the R package fdapace (Dai, Hadjipantelis, Ji, Müller, & Wang, 2017). Within this analysis, we examined the relationships between MWF as longitudinal covariates, baseline covariates (SES‐ME, child gender, birth weight, and infant feeding choice—exclusive or nonexclusive breastfeeding for the first 90 days), and longitudinal responses of cognitive functioning (ELC, VDQ, and NVDQ) from 150 to 1,000 postnatal days. Though the regression coefficients were calculated for this slightly restricted window, all scans from the complete data set spanning 65–1,481 days were utilized in the calculation to produce more reliable estimates. This is because the estimation of the regression effect at each time point through kernel smoothing requires the availability of data in a small temporal neighborhood of that time point in order to avoid boundary effects. We constructed bootstrap confidence intervals using 10,000 bootstrap samples for statistical inference. Bonferroni adjustment was used for testing the regression effects at 200, 400, 600, 800, and 1,000 days of age.

### 2.5 Whole‐brain white matter MWF

We used FCRMs to investigate the effects of white matter maturation and myelination, as measured by MWF, on each of the three composite cognitive measures, ELC, VDQ, and NVDQ. Examining first the effect of whole‐brain white matter, we used the following FCRM,

(3) $$\begin{array}{c} E\left[Y\left(t\right)|\text{covariates}\right]=\alpha \left(t\right)+\beta_{1}\left(t\right)\text{WhiteMatter}\left(t\right)+\beta_{2}\left(t\right)\text{BirthWt}+\beta_{3}\left(t\right)\text{MixedFd}\\ +\beta_{4}\left(t\right)\text{BottleFd}+\beta_{5}\left(t\right)\text{Male}+\beta_{6}\left(t\right)\mathrm{SES}, \end{array}\;$$

where *Y*(*t*) is one of the cognitive scores, MixedFd is an indicator for mixed breastmilk and formula feeding, BottleFd is an indicator of exclusive formula feeding, and Male is an indicator for a male child. Children who are exclusively breastfed have MixedFd = 0 and BottleFd = 0. As a measure of SES, maternal education level was quantified by the 7‐level scale in the Hollingshead 4‐Factor Index of SES (Hollingshead, 1975). In addition to white matter MWF as a longitudinal covariate, child gender, birth weight, and whether the child was exclusively breastfed for the first 90 days, exclusively bottle (formula) fed, or received a mixture of breast milk and formula, were included as baseline covariates.

### 2.6 Regional analysis

White matter MWF values were obtained for the whole‐brain white matter as well as in 23 individual brain regions: The body, genu, and splenium of the corpus callosum; the right and left hemisphere frontal, occipital, parietal, temporal and cerebellar white matter; corona radiata, cingulum, optic radiation, internal capsule, and superior longitudinal fasciculus. Masks for each of these regions were derived from the ICBM and JHU white matter atlases (Hua et al., 2008; Mazziotta et al., 2001). Masks were superimposed on each individual's data and mean values were calculated (Deoni et al., 2012). As previously described (Dean, O'Muircheartaigh, et al., 2014), all data were aligned using a longitudinal registration pipeline. Mask alignment was visually checked and manually edited if necessary. Mean MWF values were calculated for each masked anatomical region and used for the following analysis.

For this regional analysis, we implemented a family of simple FCRMs,

(4) $$E\left[Y\left(t\right)|X\left(t\right)\right]=\alpha \left(t\right)+\beta_{1}\left(t\right)X\left(t\right),$$

where *Y*(*t*) denotes one of NVDQ, VDQ, or ELC, and the covariate *X*(*t*) is the measure of MWF in one of the 23 individual brain regions.

### 2.7 Coefficient of determination

In classical regression modeling, the coefficient of determination, defined as the fraction of variance explained by the model, measures how well the response is linearly predicted by the covariates. We define similarly for FCRM the time‐dynamic coefficient of determination, *R*^2^(*t*), as

(5) $$R^{2}\left(t\right)=\frac{\;\mathrm{cov}\left(W\left(t\right),Y\left(t\right)\right)\prime \mathrm{var}{\left(W\left(t\right)\right)}^{-1}\;\mathrm{cov}\left(W\left(t\right),Y\left(t\right)\right)}{\;\mathrm{var}\left(Y\left(t\right)\right)}.$$

This is a direct generalization of the coefficient of determination *R*^2^ as the fraction of variance explained by a linear model to the time‐dynamic case. The measure *R*^2^(*t*) is used to quantify how well the cognitive scores were explained by measures of white matter MWF in individual brain regions as age varied. Pointwise significance for *R*^2^(*t*) was determined by the equivalent bootstrap confidence interval for β(*t*). For the time points where the whole brain white matter MWF effect was significant in model (Equation 3), significance results were adjusted for 23 brain regions to control the false‐discovery rate (FDR) by the Benjamini–Hochberg procedure (Benjamini & Hochberg, 1995).

## 3 RESULTS

### 3.1 Whole‐brain white matter MWF

**Figure 3:**
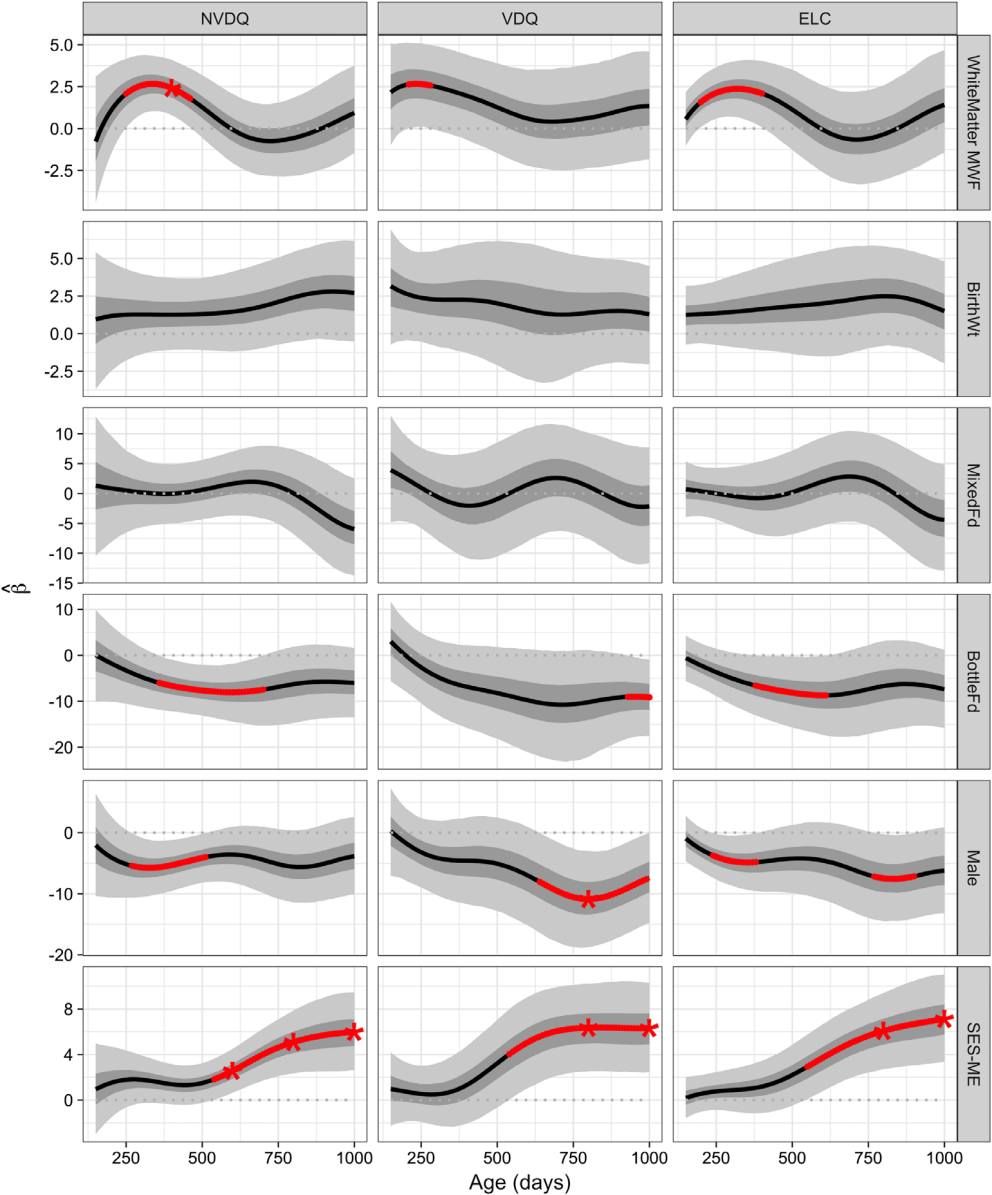
Parameter estimates for functional concurrent regression models. Each column corresponds to a model with a different cognitive response, as indicated at the top, and each row corresponds to a covariate, where the first row shows the effects of the time‐varying covariate white matter MWF and the other rows show the effects of the baseline covariates as age varies. WhiteMatter MWF and BirthWt are scaled to have unit standard deviations to facilitate comparisons. Black solid lines correspond to the regression function estimates, and dark and light gray bands correspond to 50 and 95% bootstrap confidence intervals, respectively. Where these bands do not cover 0 this corresponds to pointwise significant regression effects at the 5% level (colored in red). Significance after adjusting for multiple time points (200, 400, 600, 800, and 1,000 days) is indicated by red asterisks [Color figure can be viewed at http://wileyonlinelibrary.com]

The resulting estimates for the FCRM as given in Equation 3 and pointwise bootstrap confidence intervals for the regression coefficients are shown in Figure 3, where the columns correspond to the models for the responses NVDQ, VDQ, and ELC, respectively. Red color indicates significance at the 5% level, while asterisks indicate significance even after multiple adjustments. WhiteMatter MWF and BirthWt were scaled to have unit *SD*s in the plots to facilitate comparisons of the regression effect sizes. Significant associations after Bonferroni adjustment were white matter MWF with NVDQ at 400 days; male with VDQ at 800 days; SES‐ME with all three early learning scores at 800 and 1,000 days, and with NVDQ additionally at 600 days. Our results show that MWF is an important covariate of early cognitive development across the first 1.5 years of life, as demonstrated by the positive effect of white matter MWF on each cognitive score until approximately 500 days of age. The age range across which white matter MWF was a pointwise significant covariate was 250–450 days for nonverbal functioning (NVDQ), 260–300 days for verbal functioning (VDQ), and 200–400 days for general cognitive ability (ELC). For all three cognitive measures, maternal SES as measured by the education level had an increasing positive effect on cognitive outcomes, becoming significant after 500 days of age. Bottle feeding in contrast to early exclusive breastfeeding had a generally negative impact on cognitive maturation, pointwise significant around 500 days for ELC and NVDQ. Mixed‐feeding in contrast to breastfeeding did not have a significant effect. Being a male had a generally negative effect on cognition, pointwise significant between 250 and 500 days for NVDQ, 680–1,000 days for VDQ, and 300–800 days (marginal significance) for overall cognitive ability.

### 3.2 Regional white matter MWF

While informative, investigations of whole‐brain development with respect to cognitive maturation may mask subtle, region‐specific association given the known functional specialization of different brain regions, systems, and networks. To shed insight into regional differences in observed relationships, we examined the associations between cognitive scores and MWF values obtained from distinct brain regions and white matter pathways. It is reasonable to pursue this investigation since the whole brain white matter MWF had an association with NVDQ, which was significant at 400 days, and with VDQ and ELC, which was pointwise significant for certain periods before multiple testing adjustments. For this analysis, we considered a family of simple FCRMs as described in Equation 4.

**Figure 4:**
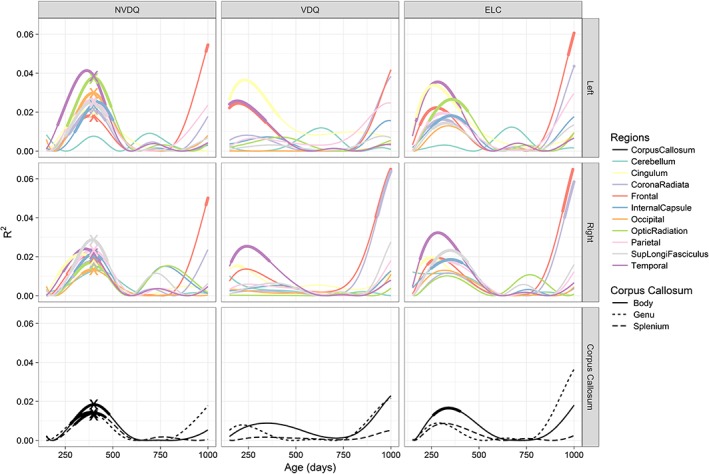
Time‐varying coefficients of determination *R* ^*2*^ *(t)* for the fits of functional concurrent regression models *E*[*Y*(*t*)|*X*(*t*)] = *α*(*t*) *+ β1*(*t*)*X*(*t*), in dependence on age *t*, where *X*(*t*) is the white matter MWF for one of the 23 individual brain regions, and *Y*(*t*) is one of the cognitive scores (NVDQ, VDQ, or ELC). The three columns (from left to right) correspond to NVDQ, VDQ, and ELC response, respectively, and the three rows (from top to bottom) correspond to the left brain, the right brain, and the corpus callosum, respectively. Bolded curve segments indicate unadjusted pointwise significance, while “×” signs indicate significant effects after controlling the FDR at the 5% level

We show in Figure 4 the results for the functional coefficients of determination *R*^2^(*t*), quantifying the degree of association with cognitive outcomes. Multiple adjustment by the Benjamini–Hochberg procedure for the 23 investigated regions was performed for NVDQ at 400 days when the whole brain white matter MWF effect was significant. In Figure 4, each column reflects one of the three cognitive scores of interest, and the rows denote the right, left, and midline hemisphere structures or regions, while bolded curve segments indicate unadjusted pointwise significance, and “×” signs indicate adjusted significance after controlling the FDR at the 0.05 level. All but the bilateral cerebellum regions were significantly associated with NVDQ at 400 days after adjustment. For each cognitive score, the *R*^2^(*t*) curves displayed a generally consistent shape, with an early peak followed by a plateau near 2 years of age. For example, most of the *R*^2^(*t*) curves corresponding to NVDQ showed a peak near 400 days of age before returning to near 0, and for bilateral frontal regions then increasing again after 750 days of age. The *R*^2^(*t*) curves for VDQ were similar but exhibited a smaller early peak magnitude and for bilateral frontal and corona radiata regions a larger increase at 750 days. However, the *R*^2^(*t*) curves for bilateral corona radiata and right frontal did not show significant early peaks for the VDQ response. The *R*^2^(*t*) curves for ELC are an approximate average of their corresponding NVDQ and VDQ curves, in line with the definition of ELC. Overall the *R*^2^(*t*) values for the NVDQ response models were larger than those for the VDQ and ELC response models, suggesting that NVDQ is more associated with white matter MWF.

The results presented in Figure 4 indicate that there is an early period during which white matter maturation, as measured by MWF, is associated with cognitive development. Exploratory observations also indicate an intervening period between approximately 500 and 750 days (1.5–2 years) of age where the association is much less pronounced, and a late period after 750 days where the association increases again for regions such as frontal and corona radiata; these results were not supported by significant *p* values, therefore are only suggestive, and will require verification in future studies. The biological interpretation and underpinning of these trends are unclear at this time.

## 4 DISCUSSION

FCRM provides a fully longitudinal nonparametric approach for the dynamic concurrent regression relationship between longitudinal processes, which can be applied to identify the time‐varying strength of associations and critical periods when these associations are significant. FCRM is able to estimate the association at arbitrary ages with sparse and irregular observations, without a priori binning the longitudinal observations or invoking shape assumptions on the longitudinal trajectories or association. In contrast, classical linear mixed effects models (Gautam, Nuñez, Narr, Kan, & Sowell, 2014) impose linear constraints on growth so cannot detect association in arbitrary time windows, and nonlinear mixed effects models (Remer et al., 2017) have only been applied to group but not individual trajectories and impose parametric shape assumptions on trajectories. In this study, FCRM is applied to study associations between longitudinal cognitive development and longitudinal white matter myelination, measured by Mullen scores and MWF, respectively, adjusting for other covariates such as gender, feeding method, birth weight, and maternal education. This longitudinal modeling approach can be widely applied to investigate the dynamic relationship between other longitudinal processes of interest in neuroimaging studies, for example, longitudinal cortical maturation or morphometry, and cognition and physical development.

Our results demonstrate a positive association between white matter MWF and emerging cognitive abilities, significant at 400 days for NVDQ as response. We also demonstrate a direct relationship between MWF and overall cognitive ability and motor and language functioning for various ages, allowing us to examine the evolution of these relationships with age and to pinpoint developmental periods when these relationships are particularly prominent. These results build on prior investigations by our group and others that not only relate brain structure to cognitive function, but also examine the association with baseline covariates.

Varying imaging acquisition parameters with age may affect derived results, as is the case for more conventional qualitative or semi‐qualitative measures such as white matter volume or density. However, as we and others have previously shown, quantitative metrics such as qT1, qT2, and MWF are much less sensitive to acquisition parameters and even different imaging hardware (RF coils, and scanner manufacturer), making the age‐varying parameters less of a concern (Deoni et al., 2012). Nonetheless, we performed an analysis where white matter maturation is measured by white matter volume (Figure S2). This additional analysis revealed no significant associations between this alternative imaging metric and cognitive functioning, indicating MWF might be preferable over volume as imaging metric for white matter development.

Investigating the relationships between whole‐brain and regional white matter MWF and cognitive development, we note an evolving trend with MWF being an important covariate from approximately 250 to 450 days of age, which is found to be significant after adjusting for multiple testing at 400 days for NVDQ. Weak and insignificant association appears to be manifest from 450 to 750 days, followed by an increase for some specific brain regions into childhood, where these observations were however not significant after adjustment, and thus are only suggestive and will require future confirmatory analysis.

Though our main analysis focused on the population normed composite scores ELC, NVDQ, and VDQ, in response to a reviewer we also compared the analysis results for the five raw and normalized Mullen subscales in order to investigate the effect of normalization. On the model level (Equation 2), any normalization of the response in FCRM would result in an equivalent model, in the sense that the existence of a regression effect (*β*(*t*) ≠ 0) and the percent of total variance explained would remain the same before and after normalization. Additional data analysis included in the Supporting Information demonstrated that the regression coefficient estimates for the raw (Figure S6) and the normalized (Figure S7) subscales had largely identical trend and similar statistical significance, where the statistical significance differed only for a few covariates within short time periods. While it is possible to normalize within our samples instead of using the population norm, this in‐sample normalization method poses a problem, namely the observations are utilized in both the estimation of the mean and the *SD* in the normalization step (Chiou, Chen, & Yang, 2014), as well as in the FCRM model [Equation A3 in the Appendix]. Additional bias may be incurred due to this two‐step procedure.

Longitudinal relationships between white matter development and cognition have been scarcely studied, which calls for future research. Results in our longitudinal analysis, while providing a detailed spatiotemporal analysis, are broadly consistent with our prior cross‐sectional reports relating MWF with overall cognitive ability (Deoni et al., 2016) and language abilities (O'Muircheartaigh et al., 2014). Deoni et al. (2016) found little association in the first year of life, diffuse and widespread associations in the second year of life, and regionally consolidated associations between 2 and 5.5 years of age. Developmentally, this is suggestive of an early period of functional onset followed by increasing specialization into and throughout childhood. Our longitudinal findings are also in agreement with prior cross‐sectional analyses identifying relationships between white matter maturation and cognition across the investigated age range. Associations have been established between white matter microstructure and working memory scores (Short et al., 2013); regional volume and Bayley scores (Shapiro et al., 2017); as well as myelination and processing speed (Chevalier et al., 2015).

Investigating the association with other baseline covariates also provides results that are consistent with, but extend, prior cross‐sectional findings. For example, disparities in SES, an umbrella term that incorporates factors including parental education level, family income, and social standing, have consistently been linked to differences in child cognitive ability, as well as social and educational outcomes (Bradley & Corwyn, 2002; Hackman & Farah, 2009; Noble, Houston, Kan, & Sowell, 2012; Turkheimer, Haley, Waldron, D'Onofrio, & Gottesman, 2003). Thus, it is unsurprising that our results show maternal education level to be a significant covariate for child cognitive functioning. However, prior reports have traditionally been cross‐sectional and, thus, the evolution of the relationship between maternal education and cognitive ability with child age throughout early childhood has not been reported.

Our finding that the effect of maternal education is the strongest among all covariates, increases with age, becoming significant by 1.5–2 years of age may be suggestive of an early “window of opportunity” during which interventions may be most effective at minimizing later disparities (Campbell & Ramey, 1994). As was pointed out by a reviewer, other factors that might account for this effect include the genetic component for cognition reflected in maternal education which has been found to increase its effect on general cognitive ability through childhood (Haworth et al., 2010), as well as the improved reliability of the response Mullen scales (Mullen, 1995). SES was chosen to be measured by maternal education because maternal education has previously been shown to strongly correlate with child physical and cognitive health and development (Bradley & Corwyn, 2002; Desai & Alva, 1998; Dollaghan et al., 1999). The maternal education scale of HI is rather stable, unlike the occupational scale, and is the component most associated with the full HI score (Bornstein, Hahn, Suwalsky, & Haynes, 2003).

Results presented here extend previous findings of brain structural disparities in breast versus formula‐fed children (Deoni, Dean, et al., 2013; Isaacs et al., 2010; Kafouri et al., 2013; Luby et al., 2016; Ou et al., 2014) to cognitive differences, with suggestive negative associations between cognitive functioning and exclusive formula‐feeding relative to breastfeeding, although these associations were insignificant after adjustment. Of note, no significant difference in cognition was found in association with mixed breast and formula feeding and exclusive breastfeeding, thus providing no evidence against the previously reported finding that even limited breastfeeding can positively influence early neurodevelopment relative to exclusive formula (Borra, Iacovou, & Sevilla, 2012).

Our results also suggest differential typical development with respect to gender (Figures 2 and 3) in addition to SES‐ME and early nutrition. Males tended to have higher white matter MWF than females between 1.5 and 2.5 years of age, but lower cognitive ability throughout the age window we investigated, where the gender effect is found to be significant for VDQ at 800 days, and pointwise significant for NVDQ (250–500 days) and ELC (250–400 and 750–900 days). These findings mirror known sexual dimorphism in trajectories of brain structure throughout childhood (Lenroot et al., 2007) and adolescence (De Bellis et al., 2001). Further, these findings may reflect other early life influences not specifically investigated here. For example, early life stress has been shown to more greatly affect male compared to female brain structures (Shors, Chua, & Falduto, 2001; Westenbroek, Boer Den, & Veenhuis, 2004), and there exist significant gender differences in the prevalence of developmental disorders, including autism (Wing, 1981), attention deficit and hyperactivity disorder (Gaub & Carlson, 1997; Szatmari, Offord, & Boyle, 1989), as well as reading disabilities (Hawke, Olson, Willcut, Wadsworth, & DeFries, 2009). Subgroup analysis in males and females separately (Figures S3 and S4) revealed similar association patterns in both genders without discernible gender interaction effects.

Examining the overall relationship trends (Figure 4) we note expected regional differences in the associations between brain MWF and verbal, nonverbal, and overall cognitive ability, an early period of positive association (200–500 days) when MWF is more strongly associated with cognitive scores, and for bilateral frontal and corona radiata regions a late period (>750 days) of increasing association, in comparison to an intermediary period when the association is overall low and statistically insignificant. Though generally weak, the associations between NVDQ and most brain regions at the early peak of 400 days are found to be significant after adjustment. This shows that structural development in individual brain regions is associated with cognitive outcomes, though the former is a weaker factor than SES or maternal education. A nonparametric bootstrap was implemented to investigate the significance of covariates, which is a more conservative and less biased approach compared to parametric tests based on mixed effects models, and this may have led to less significance of the associations.

The brain region for which MWF is observed to be most correlated with overall cognitive ability (ELC) for the early period is temporal lobe white matter, and optic radiation for nonverbal (NVDQ); while for the late period after 750 days, ELC is the most associated with the frontal white matter. While these observations do not retain statistical significance after multiple adjustments, they nevertheless align with the known spatiotemporal pattern of myelination, and with established regional function specificity. NVDQ is a composite of fine motor and visual functioning and, thus, the involvement of the optic paths (Berman et al., 2009) is not surprising. Further, the optic radiation connects the lateral geniculate nucleus to the occipital pole through the temporal loop (Catani, Jones, Donato, & Ffytche, 2003), which may explain the role of the temporal white matter as an early predictor of ELC, as for children less than 1 year of age, ELC is strongly based on visual and motor functioning. In older children, the frontal white matter and associated white matter pathways are associated with aspects of executive functioning, including attention and working memory (Alvarez & Emory, 2006; Miyake et al., 2000; Prabhakaran, Narayanan, Zhao, & Gabrieli, 2000), which are important contributors to general cognitive functioning (Ardila, Pineda, & Rosselli, 2000).

The nature of the time‐dynamic relationships between MWF and cognition remains less clear for the period between ~1.5 and 2 years of age, when the association between these measures is at a low level. Further investigation into this time period using more sophisticated imaging measures is therefore warranted. For example, there could be a trade‐off between myelination and neuronal density throughout this age period that is masked by our use of mean MWF measures. The use of non‐Gaussian diffusion models, such as NODDI (Zhang, Schneider, Wheeler‐Kingshott, & Alexander, 2012) may allow us to investigate neuronal density. Further, this information can be combined with mcDESPOT to yield the myelin g‐ratio (Dean et al., 2016). These more fine‐grained measures of tissue structure may provide additional insight into the neuroanatomical changes across this age window that are associated with cognitive development. Additional explanations may include high variability in quantifiable developmental assessment as reflected in VDQ and NVDQ between 500–750 days, where low association was observed.

The FCRM is able to handle sparse and irregular observations, provided the pooled observations are dense within the investigated time period. Though our complete data set included scans up to 1,481 days, fewer scans were available after 900 days (Figure 1) and thus we chose the window of investigation to be from 150 to 1,000 days after birth. The complete data set was utilized in estimating the regression effects within this time window in order to alleviate boundary effects in kernel smoothing (see the Appendix). An additional analysis for the concurrent regression effects up to the first 1,400 days (Figure S1) shows that the coefficient estimates suffered from larger variances in a later period, especially after 1,200 days, and none of the regression effects were significant after 1,000 days after multiple adjustments.

FCRM has been proven effective and flexible in modeling the concurrent regression relationship between longitudinal responses and covariates of time‐varying or time‐invariant nature. The flexibility of FCRM for longitudinal studies is reflected in three ways: First, it is able to tackle sparse and irregular designs and to include subjects who have as few as one measurement; second, FCRM allows one to pinpoint the critical periods where the longitudinal association between a covariate and the response is the most salient; and third, baseline and longitudinal covariates can be appropriately controlled. The estimation procedure by kernel smoothing improves efficiency of the estimates, as compared to a simple sliding window approach. In future work, refined versions of FCRM may be applied to create a voxelwise spatiotemporal map of association between myelination and cognitive function.

## 5 CONCLUSION

Early neurodevelopment is a dynamic process during which brain structure and function symbiotically evolve together. Here, we have sought to investigate this evolving structure–function relationship by applying FCRMs to longitudinal neuroimaging and neurocognitive data for the first time. FCRM is flexible enough to quantify arbitrary dynamic associations between time‐evolving processes at different ages and can handle genuinely sparse and irregular data, overcoming the limitations of cross‐sectional or parametric approaches. Results reveal a more temporally dynamic relationship pattern than previously presented, characterized by an early period (200–500 days) of general development, during which there is a relatively strong association between brain myelination (measured by MWF) and cognitive ability. Our exploratory results also suggest a late period (>750 days) of increased association for specific regions, an observation that needs future confirmatory analysis. Investigating the relationship of additional baseline covariates, we find that the association between maternal education (as a proxy for SES) and cognition is not only positive but furthermore increases with child age. These results provide new insight into the emerging patterns of brain and cognitive development and support the further use of FCRM for investigating these evolving relationships that could be directly applied to other longitudinal child development data sets.

## Supporting information


**Appendix S1**: Supporting Information

## Data Availability

Data available on request due to privacy/ethical restrictions.

## APPENDIX 1

### ESTIMATION

By standard least squares theory, the regression parameters can be written as

(A1) $$\beta \left(t\right)=\mathrm{var}{\left(W\left(t\right)\right)}^{-1}\;\mathrm{cov}\left(W\left(t\right),Y\left(t\right)\right),$$

and

(A2) $$\alpha \left(t\right)=\mu_{Y}\left(t\right)-\mu_{W}{\left(t\right)}^{\prime }\beta \left(t\right),$$

where *μ*_*Y*_(*t*) = *E*(*Y*(*t*)) is the expected value of the response at age *t*, and $\mu_{W}\left(t\right)=E\left(W\left(t\right)\right)=\left[\mu_{X_{1}}\left(t\right),\ldots ,\mu_{X_{p}}\left(t\right),E\left(Z_{1}\right),\ldots ,E\left(Z_{q}\right)\right]\prime$ is the expected values of the covariates at age *t*.

Since the observations are sparse and irregular, it is not viable to estimate the regression coefficients cross‐sectionally. Following Şentürk and Nguyen (2011), we pooled the information across all subjects and obtained estimates utilizing all measurements. In view of Equations 5, A1, A2, in order to estimate *α*(*t*), *β*(*t*), and *R*^2^(*t*), we need to obtain estimates for the mean and the covariance functions using the pooled data and then use the corresponding plug‐in estimates for Equations 5, A1, A2. That is,

(A3) β^t=var^Wt−1cov^WtYt,

(A4) α^t=μ^Yt−μ^Xt′β^t

and

(A5) $${\hat{R}}^{2}\left(t\right)=\frac{\hat{\mathrm{cov}}\left(W\left(t\right),Y\left(t\right)\right)\prime \hat{\;\mathrm{var}}{\left(W\left(t\right)\right)}^{-1}\hat{\mathrm{cov}}\left(W\left(t\right),Y\left(t\right)\right)}{\hat{\mathrm{var}}\left(Y\left(t\right)\right)}.$$

The following describes the estimation procedure for the mean and the covariance functions, regression coefficients, and *R*^2^(*t*). For the *j*th visit of the *i*th subject at time *t*_ij_, denote our observations as (*X*_1ij_,  … , *X*_pij_, *Z*_1i_,  … , *Z*_qi_, *Y*_ij_) for *i* = 1, … , *n* and *j* = 1, … , *n*_i_, where *n* is the number of subjects, and *n*_*i*_ is the number of measurements per subject. Here *Z*_1i_, … , *Z*_*q*i_ are the *q* baseline covariates examined, and *X*_kij_ = *X*_ki_(*t*_ij_), *Y*_ij_ = *Y*_i_(*t*_ij_) are longitudinal observations for the *k*th covariate and the response at time *t*_ij_, for *k* = 1, … , *p*, where *p* is the number of longitudinal covariates examined. We used the following procedure for estimating *μ*_**W**_(*t*), *μ*_Y_(*t*), var(**W**(*t*)) and cov(**W**(*t*), *Y*(*t*)) based on smoothing, similar to the scheme proposed by Yao, Müller, and Wang (2005) and Şentürk and Nguyen (2011):

1. For the *l*th baseline covariates *Z*_l_, we estimated its mean and covariance by the sample mean and covariance of the observations ${\left\{Z_{\mathrm{li}}\right\}}_{i=1}^{n}$.
2. For the *k*th longitudinal covariates *X*_k_(*t*) or the response *Y*(*t*), we estimated the mean function by smoothing the pooled scatter plot data tijXkij∣i=1n∣j=1ni or tijYij∣i=1n∣j=1ni using kernel local linear smoothers and obtained the mean function estimate ${\hat{\mu }}_{X_{k}}\left(t\right)$ or ${\hat{\mu }}_{Y}\left(t\right)$.
3. We then obtained the raw auto‐ and cross‐covariances as follows, regarding the response *Y(t)* the same as a longitudinal covariate:

(A6) $$G_{X_{k},Z_{l}}\left(t_{\mathrm{ij}}\right)=\left(X_{\mathrm{kij}}-{\hat{\mu }}_{X_{k}}\left(t_{\mathrm{ij}}\right)\right)\left(Z_{\mathrm{li}}-{\bar{Z}}_{l}\right),$$

(A7) $$G_{X_{k},X_{m}}\left(t_{\mathrm{ij}},t_{\mathrm{ij}\prime }\right)=\left(X_{\mathrm{kij}}-{\hat{\mu }}_{X_{k}}\left(t_{\mathrm{ij}}\right)\right)\left(X_{\mathrm{mi}j^{\prime }}-{\hat{\mu }}_{X_{m}}\left(t_{ij^{\prime }}\right)\right),$$

for *j*, *j*′  = 1, … , *n*_i_ and *i* = 1, … , *n*, where *X*_k_ and *X*_m_ are two arbitrary longitudinal covariates (or the response) and ${\bar{Z}}_{l}$ is the sample mean of the *l*th baseline covariate.

1. Next, we obtained the smoothed auto‐ and cross‐covariance estimates by applying kernel local linear smoothers on the raw covariances. For estimating the cross‐covariances cov(*X*_*k*_(*t*), *Z*_*l*_) between a longitudinal process *X*_*k*_(*t*) and a baseline covariate *Z*_*l*_ we fed raw covariances tijGXk,Zltij∣i=1n∣j=1ni to a one‐dimensional smoother. For estimating cross‐covariances cov(*X*_k_(*t*), *X*_m_(*t*)) between two longitudinal covariates *X*_k_ and *X*_m_, contrary to Şentürk and Müller (2010), we used a one‐dimensional smoother again, noting that Equations 5, A1, A2 depend only on the auto‐ and cross‐covariances of two processes evaluated at the same time‐point *t*. We took advantage of this fact and obtained $\;\hat{\mathrm{cov}}\left(X_{k}\left(t\right),X_{m}\left(t\right)\right)$ by applying one‐dimensional kernel local smoothers on the raw covariances tijGXk,Xmtijtij∣i=1n∣j=1ni.

The detailed forms for the one‐dimensional kernel local linear smoothers are described next. To estimate the mean function *μ*_X_(*t*) of a stochastic process *X*(*t*), for which we make use of the observations tijXij∣i=1n∣j=1ni, we define the kernel local linear estimate for *μ*_*X*_(*t*) as ${\hat{\mu }}_{X}\left(t\right)={\hat{\beta }}_{0}$, where

(A8) $$\left({\hat{\beta }}_{0},{\hat{\beta }}_{1}\right)=\underset{\beta_{0},\beta_{1}}{\arg\;\min}\sum_{i=1}^{n}\sum_{j=1}^{n_{i}}K\left(\frac{t_{\mathrm{ij}}-t}{h}\right){\left[X_{\mathrm{ij}}-\beta_{0}-\beta_{1}\left(t-t_{\mathrm{ij}}\right)\right]}^{2},$$

*h* > 0 is a bandwidth, and *K*(·) is a kernel function. Similarly, we used the same smoother to estimate cov(*X*_*k*_(*t*), *Z*_*l*_) from tijGXk,Zltij∣i=1n∣j=1ni and cov(*X*_k_(*t*), *X*_m_(*t*)) from tijGXk,Xmtijtij∣i=1n∣j=1ni. For all kernel local smoothing, we used the Gaussian kernel with bandwidth equal to 150 days.

For numeric covariates, the sample means and standard deviations are shown. Education level is on a 7‐point scale determined by Hollingshead four‐factor index (Hollingshead, 1975), in which a six means standard college or university graduation.

Abbreviations: ELC, early learning composite; NVDQ, nonverbal development quotient; VDQ, verbal development quotient.
