# Supplementary material for: Longitudinal associations between white matter maturation and cognitive development across early childhood
Source: Hum Brain Mapp. 2019 Jun 12;40(14):4130–45. doi: 10.1002/hbm.24690 (PMC6771612; doi:10.1002/hbm.24690)
Supplement: Supplementary file 1 — Appendix S1: Supporting Information [file HBM-40-4130-s001.docx]

**Supplementary Materials for**

**Longitudinal Associations Between White Matter Maturation and Cognitive Development Across Early Childhood**

**The Choice of the Window of Investigation**

Our functional concurrent regression models (FCRM) are able to handle sparse and irregular observations given the pooled observations are dense within the investigated time period. Since fewer scans were available after 900 days (**Figure 1**), our main analysis was based on the more reliable estimates for the window of investigation from 150 to 1000 days after birth. Data after 1000 days were still included in the inference of regression effects before 1000 days for the purpose of reducing boundary effects in kernel smoothing (see the Appendix).

We performed an additional analysis for the concurrent regression effects up to the first 1400 days after birth as shown in **Figure S1**. While the regression coefficients for first 1000 days were identical to those in the main analysis (**Figure 3**), the estimates for a later period (especially for after 1200 days) appeared less stable, as indicated by the widening confidence intervals (e.g., for white matter MWF). None of the regression coefficient were significant after 1000 days after multiple adjustments. This further justifies the examination of only the first three years of age for modeling the longitudinal association between white matter maturation and cognitive performance.

**White Matter Volume as the White Matter Imaging Metric**

We investigated the associations between cognition and the whole brain white matter volume, where the latter serves as an alternative imaging metric to the white matter myelin water fraction (MWF). The following model were fitted

$$\begin{matrix} E\left[ Y\left( t \right) | \mathrm{covariates} \right]=\alpha\left( t \right)+\beta_{1}\left( t \right)\mathrm{WhiteMatterVolume}\left( t \right)+\beta_{2}\left( t \right)BirthWt+\beta_{3}\left( t \right)\mathrm{MixedFd} \\ +\beta_{4}\left( t \right)BottleFd+\beta_{5}\left( t \right)Male+\beta_{6}\left( t \right)\mathrm{SES} \end{matrix}$$

with the estimated concurrent regression coefficients displayed in **Figure S2**. The associations between white matter volume and the cognitive response were insignificant for all three early learning scales, while the effects of the time-invariant covariates were almost identical to those in **Figure 3**. This indicates that the MWF is the preferred imaging metric over volume for characterizing white matter maturation in a young pediatric population.

**Subgroup analysis for Males and Females**

In order to examine whether gender differences exist in the associations between MWF and cognitive measurements, we considered the model

$$\begin{matrix} E\left[ Y\left( t \right) | \mathrm{covariates} \right]=\alpha\left( t \right)+\beta_{1}\left( t \right)\mathrm{WhiteMatterVolume}\left( t \right)+\beta_{2}\left( t \right)BirthWt+\beta_{3}\left( t \right)\mathrm{MixedFd} \\ +\beta_{4}\left( t \right)BottleFd+\beta_{5}\left( t \right)\mathrm{SES} \end{matrix}$$

for males and females separately. Results for males and females are displayed in **Figure S3** and **Figure S4**, respectively. The signs of the coefficient estimates for males and females agreed in general, concurring to the results found by combining the two genders (**Figure 3**). In particular, for both genders the association between white matter MWF and NVDQ/ELC scores peaked in an early period (250-500 days), and SES-MS had an increased relationship with cognitive outcomes. Fewer significant associations were found for females as compared to males, potentially due to the smaller sample size (90 vs 120, see **Table 1**) or smaller effect sizes for females. The estimated regression effects of white matter MWF on NVDQ were overall smaller for females as compared to those for males, indicating the possibility of an interaction effect between gender and white matter MWF on non-verbal cognition, though no statistical significance was established.

**Mullen Subscales and Normalization**

Mullen scales ELC, NVDQ, and VDQ are population normalized measures with mean 100 and standard deviation of 15 at each age. In response to a reviewer, we demonstrate in **Figure 2** that the means of the Mullen scales in our sample fell within the population norm for most of the ages since the 95% pointwise confidence intervals largely covered 100, while our samples had mean NVDQ slightly above population average in the first 500 days only. The deviation of our sample from the population average may prompt reconsideration of the normalization method, but we argue that this is not necessary for our FRCM.

On the model level (equation [2]), normalizing the covariates or the response in the FCRM results in an equivalent model, in the sense that the existence of a regression effect ($\beta(t)\neq0$) and the percent of total variance explained by the covariates remains the same before and after normalization. Only the magnitude of $\beta\left( t \right)$ may change after normalization due to scaling. Therefore, given a large enough number of individuals, even an inappropriately chosen normalization does not influence the inference of the regression effects.

Additional analyses were also performed to investigate the effect of normalization in our cohort of a moderate number of samples. Responses considered were the raw (unnormalized) and the population normalized Mullen subscales consisting of gross and fine motor function, visual reception, and expressive and receptive language. These subscales were considered rather than the ELC, NVDQ, and VDQ because the latter are themselves calculated from normalized scales. Raw subscales demonstrated consistent increasing patterns over age (upper panels, **Figure S5**), while the normalized ones centered around the population average 50 (lower panels, **Figure S5**).

Regression coefficient estimates for the raw and the normalized subscales are displayed in **Figure S6** and **Figure S7**, respectively. Overall, the trends demonstrated by the time-varying regression coefficients were largely identical for the raw and the normalized subscales. The statistical significance was nearly similar for both responses, and only a few covariates differed in the significance level within short time periods. This comparison validates that our FCRM is minimally affected by the normalization method for the Mullen scales.

While it is possible to normalize within our samples instead of using the population norm, this in-sample normalization method poses a problem, namely the observations would be utilized for both the estimation of the mean and the standard deviation in the normalization step (Chiou et al 2014), as well as for the FCRM model ([A3] in the Appendix). Additional bias may be incurred due to this two-step procedure.


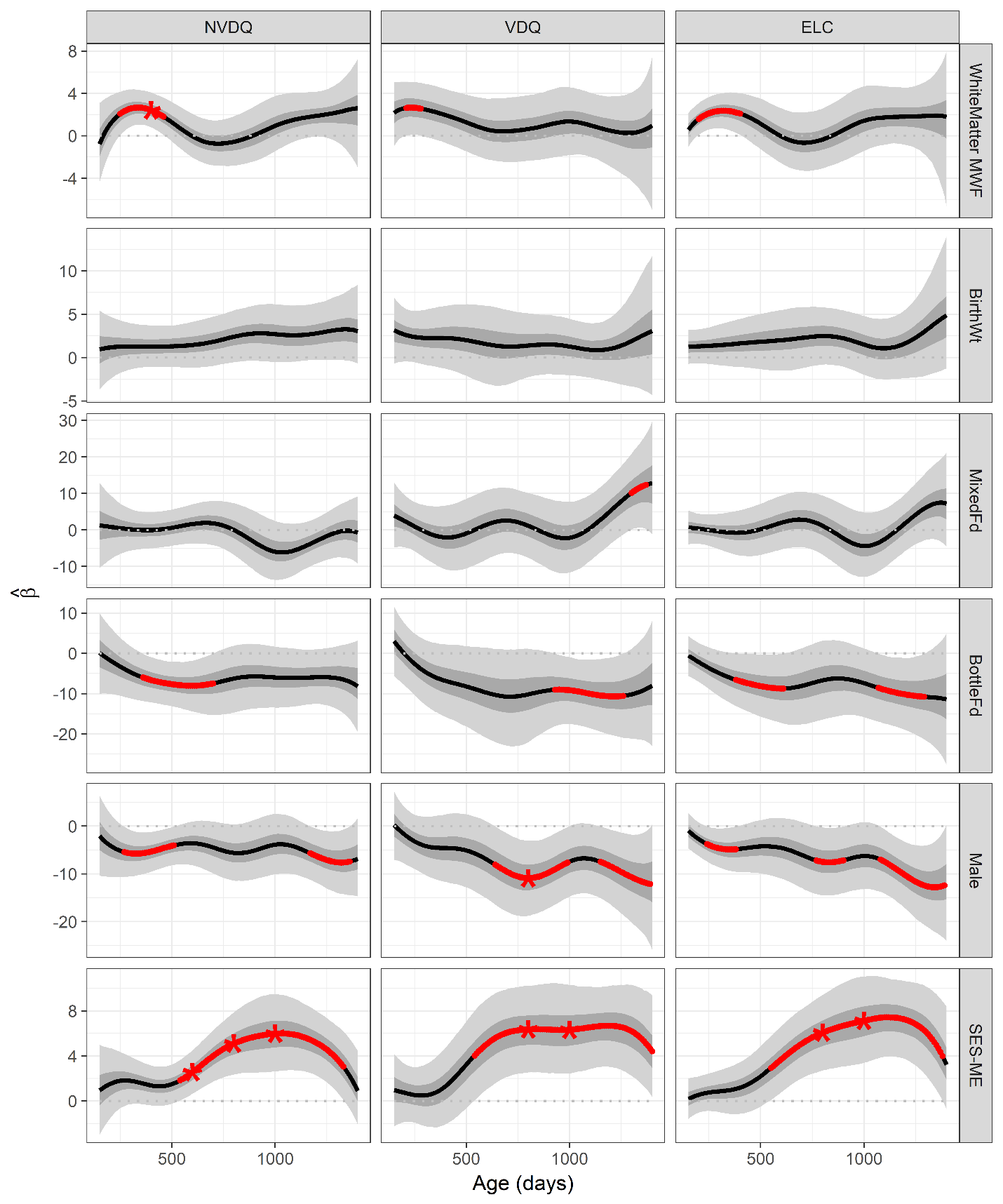


**Figure S1** Concurrent regression coefficients between covariates and cognitive responses from 150 to 1400 days after birth. Each column corresponds to a model with a different cognitive response, as indicated at the top, and each row corresponds to a covariate, where the first row shows the effects of the time-varying covariate white matter MWF and the other rows show the effects of the baseline covariates as age varies. WhiteMatter MWF and BirthWt are scaled to have unit standard deviations to facilitate comparisons. Black solid lines correspond to the regression function estimates, and dark and light gray bands correspond to 50% and 95% bootstrap confidence intervals. Where these bands do not cover 0 this corresponds to pointwise significant regression effect at 5% level (colored in red). Significance after adjusting for multiple time points (200, 400, 600, 800, 1000, 1200, and 1400 days) is indicated by red asterisks.
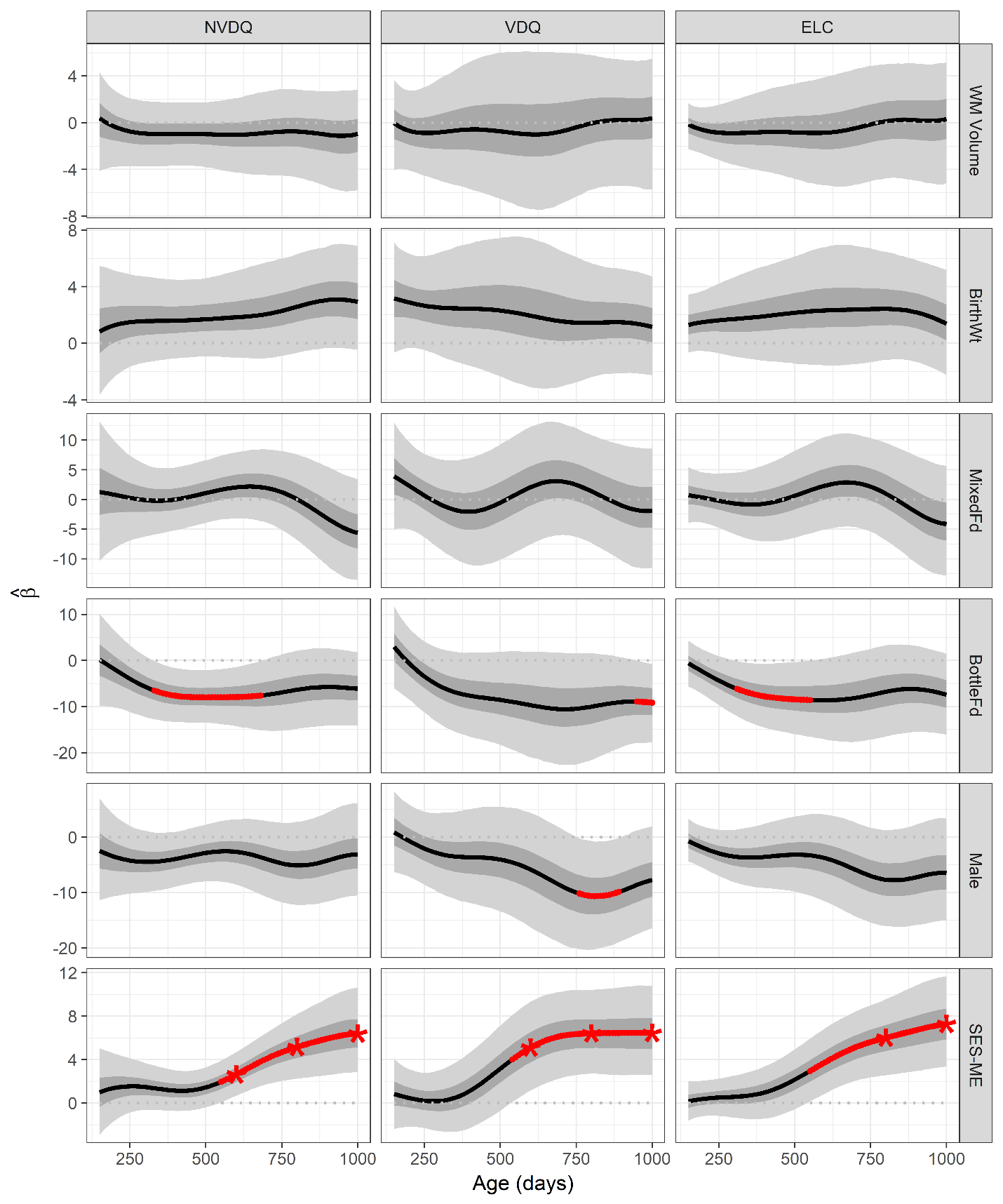
**Figure S2** Parameter estimates for fitted functional concurrent regression models with white matter volume as the imaging metric. Figure legends were identical to those in **Figure 3**. In contrast to white matter MWF as the imaging metric (**Figure 3**), white matter volume is not significantly associated with any of the VDQ, NVDQ, and ELC scores, while other non-imaging covariates had similar coefficient estimates.


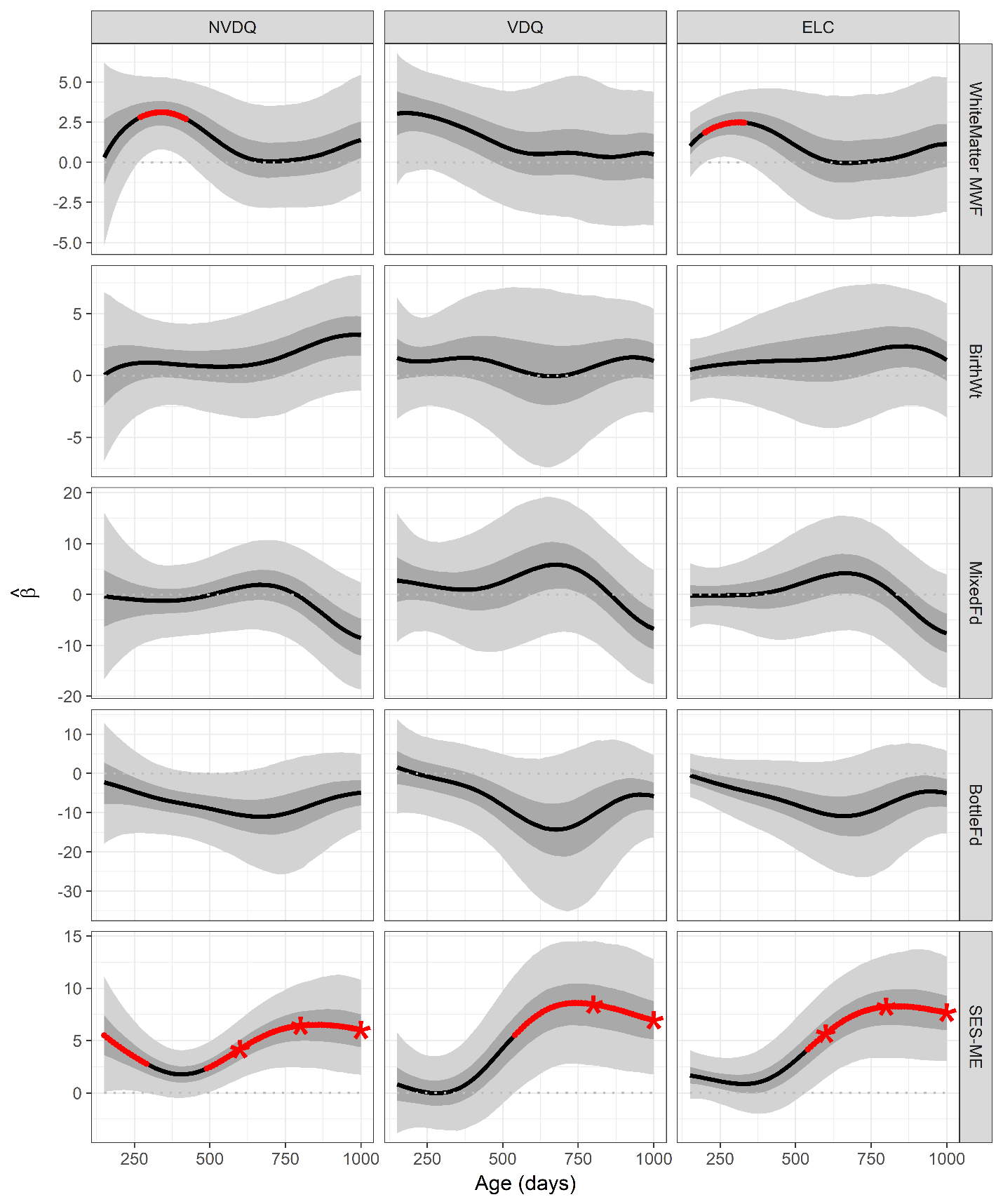


**Figure S3** Parameter estimates for fitted functional concurrent regression models with white matter volume for males only. Figure legends were identical to those in **Figure 3**.


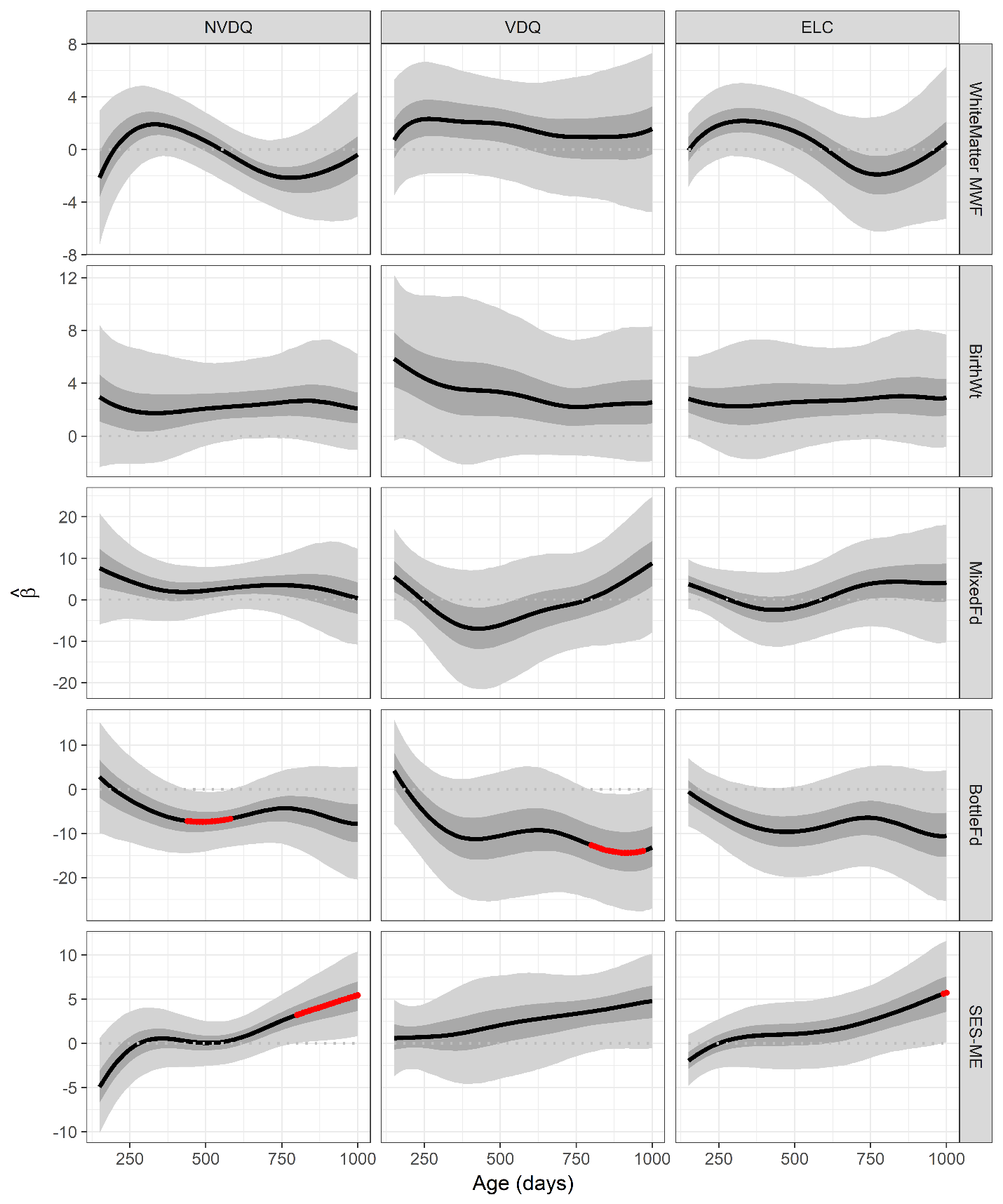


**Figure S4** Parameter estimates for fitted functional concurrent regression models with white matter volume for females only. Figure legends were identical to those in **Figure 3**.


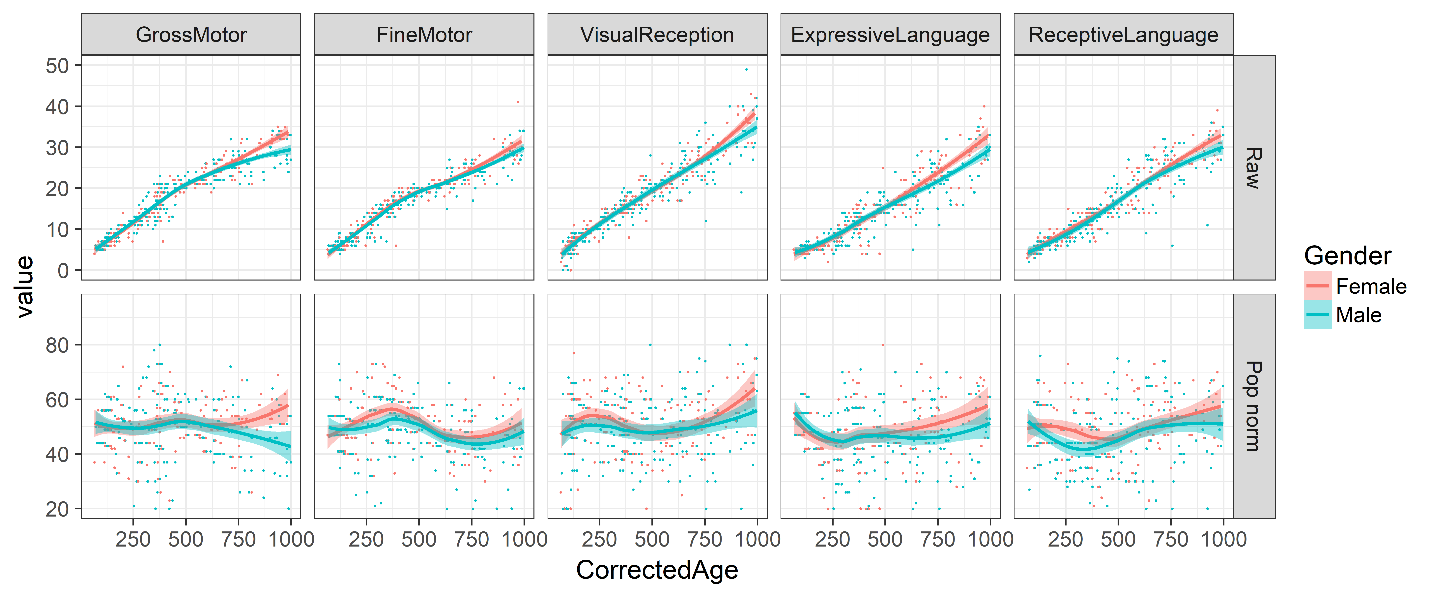


**Figure S5** Longitudinal measurements of Mullen subscales. The solid curves are the estimated mean curves over time by local quadratic smoothing, and the shaded bands are 95% confidence intervals.


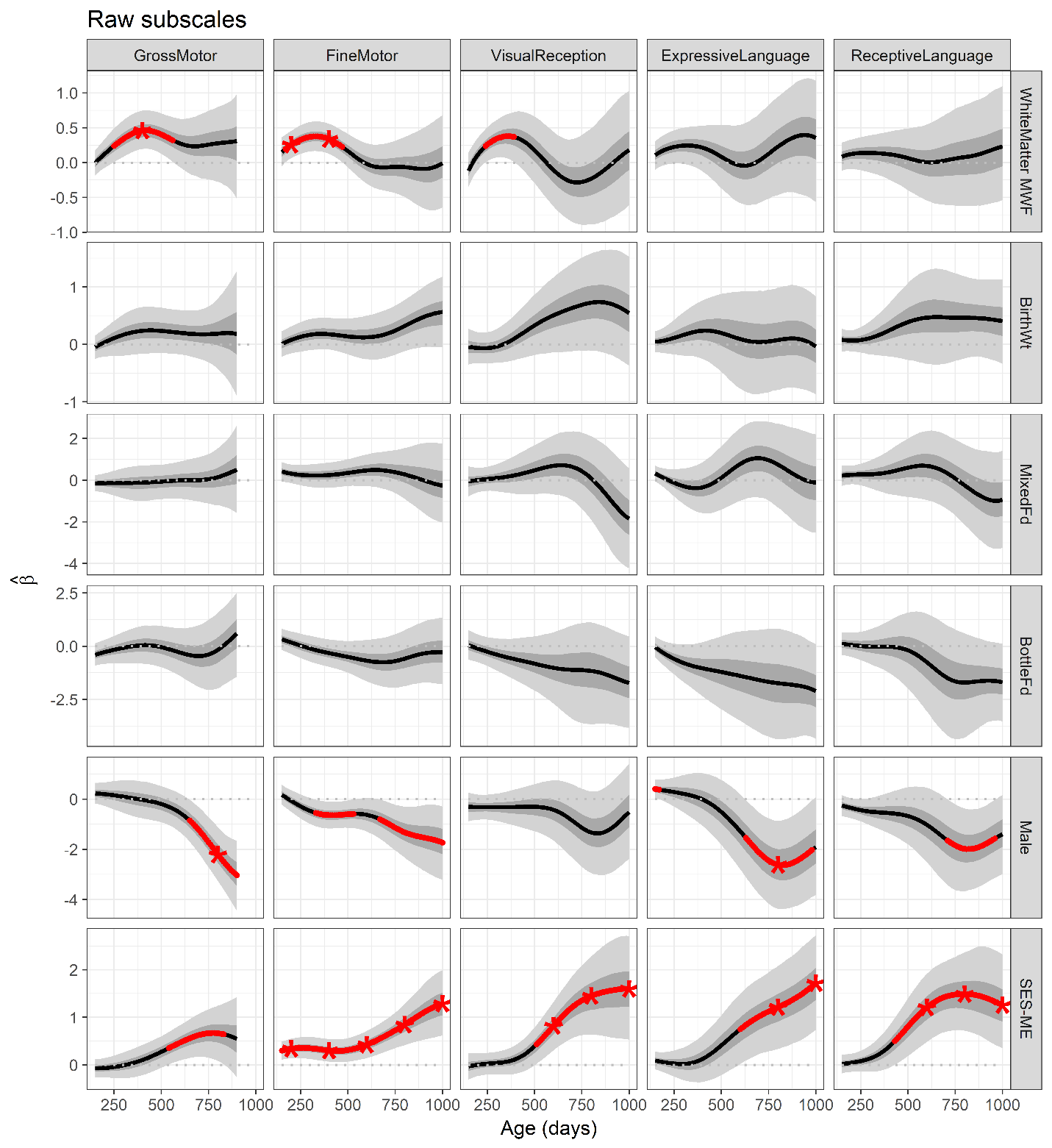
**Figure S6** Parameter estimates for functional concurrent regression models, where the responses are the raw Mullen subscales. Each column corresponds to a different raw Mullen subscale, and each row corresponds to a covariate. WhiteMatter MWF and BirthWt are scaled to have unit standard deviations to facilitate comparisons. Black solid lines correspond to the regression function estimates, and dark and light gray bands correspond to 50% and 95% bootstrap confidence intervals. Where these bands do not cover 0 this corresponds to pointwise significant regression effects at the 5% level (colored in red). Significance after adjusting for multiple time points (200, 400, 600, 800, and 1000 days) is indicated by red asterisks.
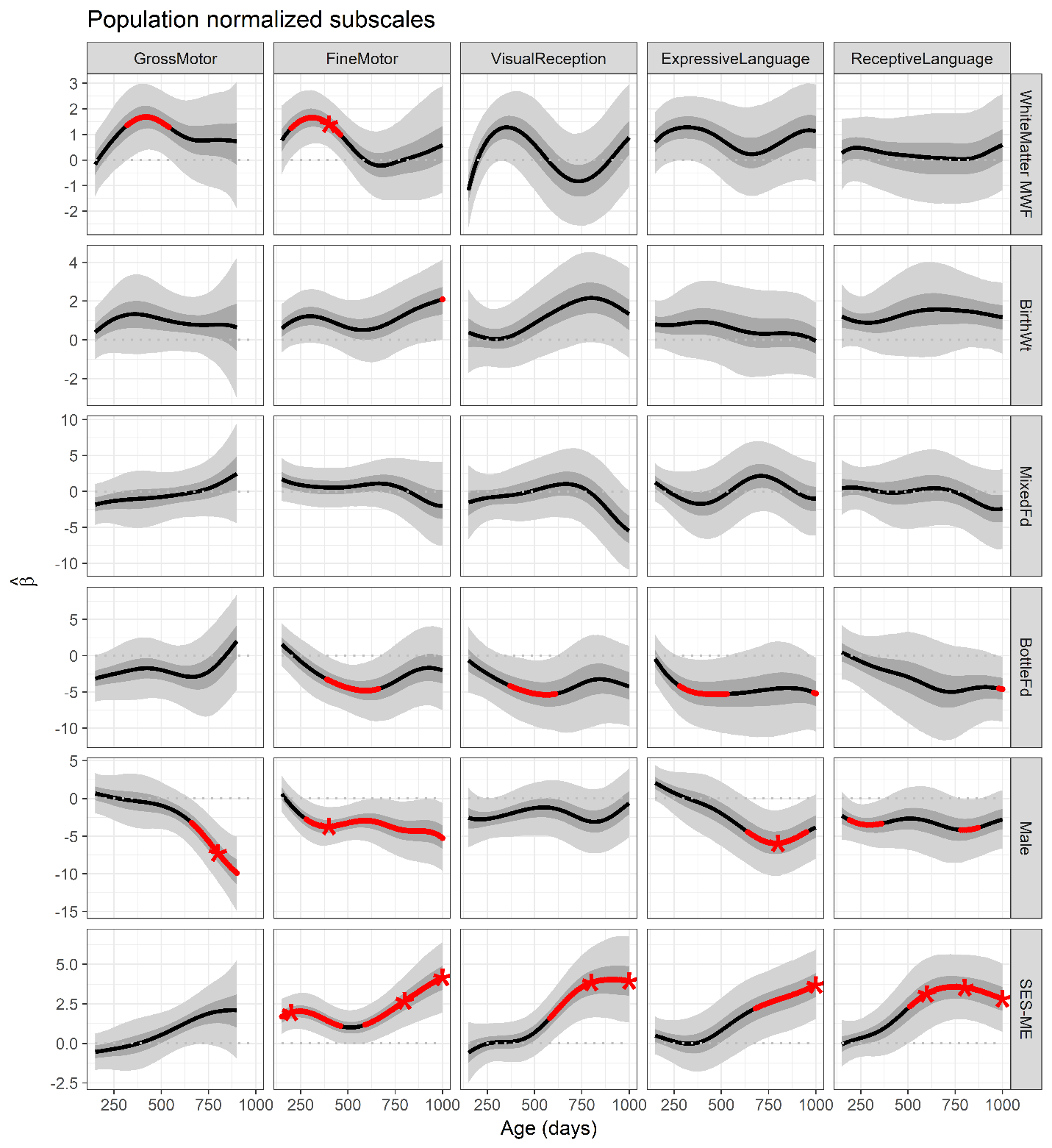
**Figure S7** Parameter estimates for functional concurrent regression models, where the responses are the population normalized Mullen subscales. Each column corresponds to a different raw Mullen subscale, and each row corresponds to a covariate. WhiteMatter MWF and BirthWt are scaled to have unit standard deviations to facilitate comparisons. Black solid lines correspond to the regression function estimates, and dark and light gray bands correspond to 50% and 95% bootstrap confidence intervals. Where these bands do not cover 0 this corresponds to pointwise significant regression effects at the 5% level (colored in red). Significance after adjusting for multiple time points (200, 400, 600, 800, and 1000 days) is indicated by red asterisks.

**REFERENCES**

Chiou JM, Chen YT, Yang YF. Multivariate functional principal component analysis: A normalization approach. Statistica Sinica. 2014 Oct 1:1571-96.
